# ICTV Virus Taxonomy Profile: *Avsunviroidae*

**DOI:** 10.1099/jgv.0.001045

**Published:** 2018-03-26

**Authors:** Francesco Di Serio, Shi-Fang Li, Jaroslav Matoušek, Robert A. Owens, Vicente Pallás, John W. Randles, Teruo Sano, Jacobus Th. J. Verhoeven, Georgios Vidalakis, Ricardo Flores, ICTV Report Consortium

**Affiliations:** ^1^​ Istituto per la Protezione Sostenibile delle Piante, Consiglio Nazionale delle Ricerche, Bari 70126, Italy; ^2^​ State Key Laboratory of Biology of Plant Diseases and Insect Pests, Institute of Plant Protection, Chinese Academy of Agricultural Sciences, Beijing 100193, PR China; ^3^​ Biology Centre of the Czech Academy of Sciences v.v.i, Institute of Plant Molecular Biology, Branišovská 31, 370 05 České Budějovice, Czech Republic; ^4^​ Molecular Plant Pathology Laboratory, US Department of Agriculture, Agricultural Research Service, Beltsville, MD 20705, USA; ^5^​ Instituto de Biología Molecular y Celular de Plantas, Universidad Politécnica de Valencia-Consejo Superior de Investigaciones Científicas, Valencia 46010, Spain; ^6^​ School of Agriculture, Food and Wine, The University of Adelaide, Waite Campus, Glen Osmond, SA 5064, Australia; ^7^​ Faculty of Agriculture and Life Science, Hirosaki University, Hirosaki 036-8561, Japan; ^8^​ Plant Protection Organization of The Netherlands, Wageningen, 6700 HC, The Netherlands; ^9^​ Department of Microbiology and Plant Pathology, University of California, Riverside, CA 92521, USA

**Keywords:** *Avsunviroidae*, ICTV, taxonomy, viroid, avocado sunblotch viroid, peach latent mosaic viroid, eggplant latent viroid

## Abstract

Members of the family *Avsunviroidae* have a single-stranded circular RNA genome that adopts a rod-like or branched conformation and can form, in the strands of either polarity, hammerhead ribozymes involved in their replication in plastids through a symmetrical RNA–RNA rolling-circle mechanism. These viroids lack the central conserved region typical of members of the family *Pospiviroidae.* The family *Avsunviroidae* includes three genera, *Avsunviroid*, *Pelamoviroid* and *Elaviroid,* with a total of four species. This is a summary of the ICTV Report on the taxonomy of the family *Avsunviroidae,* which is available at http://www.ictv.global/report/avsunviroidae.

## Genome

**Fig. 1.**
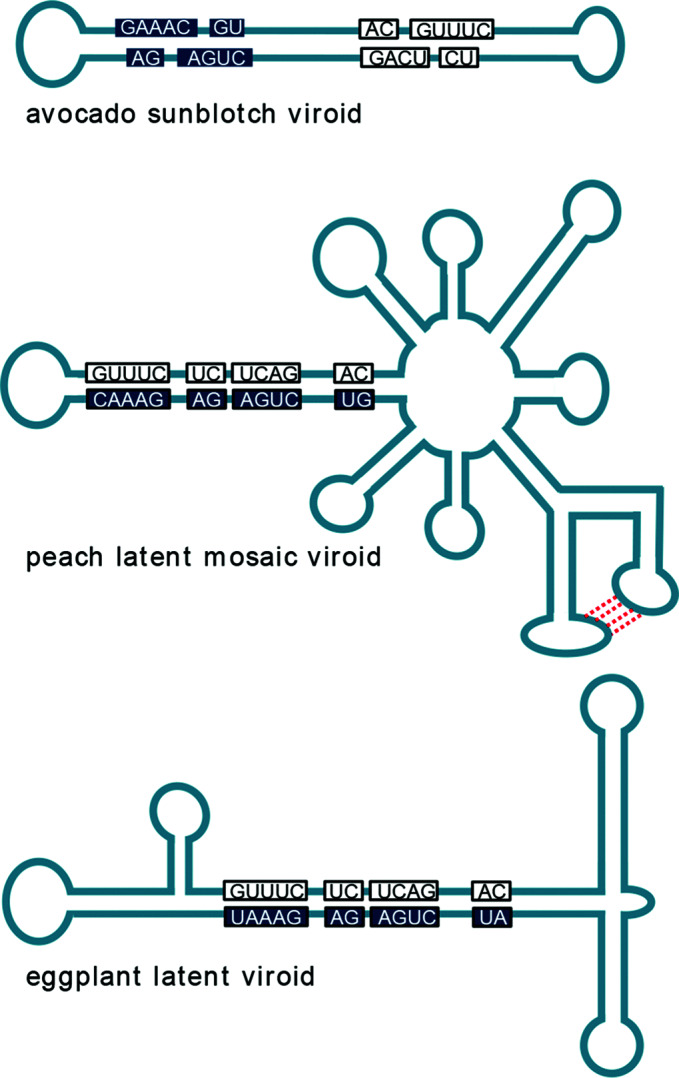
Proposed secondary structures of representative members of the family *Avsunviroidae.* Conserved nucleotides in the hammerhead catalytic core are boxed with filled and open shadings referring to ribozymes formed in the viroid (+) and (-) strand, respectively.

**Fig. 2.**
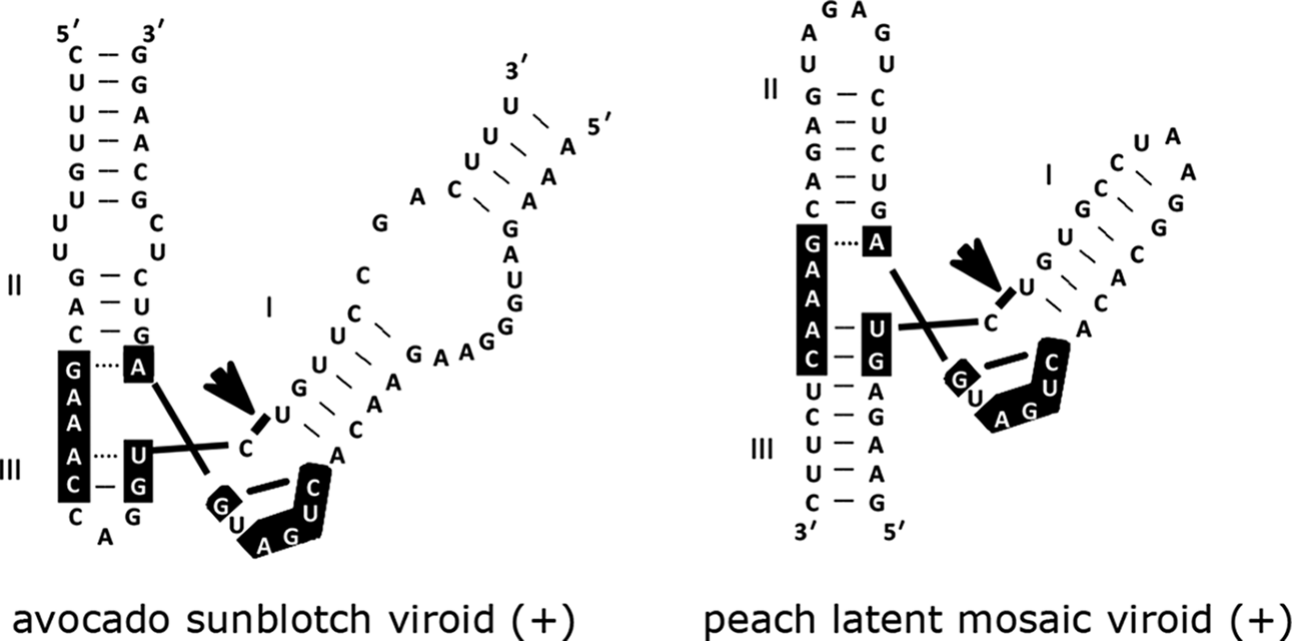
Hammerhead structures formed by the (+) strand of two viroids. Catalytic cores (black boxes), self-cleavage sites (arrows) and helices (I, II, III) are indicated. Figure modified from [7].

Members of the family *Avsunviroidae* have a circular single-stranded RNA genome of 246 to 401 nt, which may assume rod-like, quasi-rod-like or branched conformations *in silico* or *in vitro*, with indirect or direct evidence supporting similar conformations *in vivo* (Table 1, Fig. 1). Viroid G+C content is >50 % except for avocado sunblotch viroid (38 %). RNAs of (+, arbitrarily the most abundant strand *in vivo*) and (−) polarity can form active hammerhead ribozymes (Fig. 2) that are involved in replication [1, 2].

**Table 1.** Characteristics of the family *Avsunviroidae*

Typical member:	avocado sunblotch viroid (J02020), species *Avocado sunblotch viroid*, genus *Avsunviroid*
Genome	Single-stranded circular RNA of 246–401 nt that can form hammerhead ribozymes in the strands of either polarity
Replication	A nuclear-encoded plastid RNA polymerase generates complementary oligomeric RNAs that are co-transcriptionally self-cleaved by the hammerhead ribozymes. The resulting monomeric RNAs are circularized by a tRNA ligase
Host range	Plants (dicots)
Taxonomy	Three genera, collectively containing four species

## Replication

Replication takes place in plastids, mostly chloroplasts, through a symmetrical rolling-circle mechanism. A nuclear-encoded plastid RNA polymerase, conscripted to transcribe RNA templates instead of its physiological DNA template, synthesizes oligomeric viroid RNAs of both polarities. These oligomers are self-cleaved co-transcriptionally by the embedded hammerhead ribozymes, thereby generating linear monomeric RNAs that are subsequently circularized by a tRNA ligase. This enzyme, like the nuclear-encoded plastid RNA polymerase, is encoded in the nucleus and translocated into plastids.

## Taxonomy

The type of hammerhead structure, the genome G+C content and its solubility in 2 M LiCl, together with clustering in phylogenetic trees derived from whole genome sequences, are used as genus demarcation criteria.

### Avsunviroid

Members of the single species in the genus, *Avocado sunblotch viroid*, have a genome that adopts a rod-like confromation, has G+C content of 38 %, and is soluble in 2 M LiCl (Fig. 1). Hammerhead structures formed by either strand are thermodynamically unstable with a short helix III (Fig. 2); thus double-hammerhead structures may be involved in self-cleavage. Avocado is the only known natural host [3].

### Pelamoviroid

Members of the two species included in this genus (*Peach latent mosaic viroid* and *Chrysanthemum chlorotic mottle viroid*) have circular RNA genomes that are insoluble in 2 M LiCl and assume branched conformations stabilized by a kissing-loop interaction in the (+) strand (Fig. 1). Stable single-hammerhead structures (Fig. 2) mediate replication [4, 5]. Host range is restricted to the original hosts and some closely related species.

### Elaviroid

Members of the single species in this genus, *Eggplant latent viroid*, have a genome that assumes a quasi rod-like conformation (Fig. 1) and is soluble in 2 M LiCl. Both strands form stable single-hammerhead structures involved in replication. Host range is restricted to several eggplant cultivars [6].

## Resources

Full ICTV Online (10th) Report: www.ictv.global/report/avsunviroidae.
